# An Unusual Presentation of Nectrotizing Otitis Externa in an Elderly Patient

**DOI:** 10.7759/cureus.93498

**Published:** 2025-09-29

**Authors:** Iuliana Apostol-DeJong, Sana Masood, Liliana C Binzar

**Affiliations:** 1 Geriatric Medicine, James Cook University Hospital, Middlesbrough, GBR; 2 Neurology, Spitalul Clinic Județean de Urgență Sfântul Apostol Andrei, Galati, ROU

**Keywords:** cranial nerves palsy, elderly, malignant otitis externa, necrotizing otitis externa, skull base osteomyelitis

## Abstract

Necrotizing otitis externa (NOE) is a rare but life-threatening condition that can often be challenging to diagnose. In this case report, we describe a rare presentation of NOE in an 83-year-old man with subtle signs of multiple unilateral cranial nerve palsy (VII, VIII, IX, X, XII). Prior to this, he had a three-month history of bilateral otitis externa, managed with topical and oral antibiotics. NOE was confirmed on CT head, which revealed left-sided skull base osteomyelitis (SBO). A repeat CT scan revealed left sigmoid sinus thrombosis and intracerebral abscess, indicative of disease progression. He was treated with ototopic and long-term systemic antipseudomonal antibiotics but unfortunately he was too frail, with a Clinical Frailty score (CFS) of 7, for further surgical management. The disease progressed despite ward-based treatment and he sadly passed away.

## Introduction

Necrotizing otitis externa (NOE) is an infection of the external ear canal that spreads to the adjacent bone, leading to cranial osteomyelitis but also to the cranial nerves, meninges and vessels, leading to “malignant” external otitis. "Malignant otitis externa" was phrased in 1963 by Hahn and Chandler, when they reported five cases of the disease which spread from the ear to the temporal bone causing necrosis and further extension to the brain. It is not cancerous but due to its detrimental effects, it was given the name of malignant, as it carries a high risk of mortality and morbidity [1].

Due to a variety of clinical presentations, the diagnosis can be delayed with significant morbidity and mortality [2]. The disease commonly affects diabetics, the elderly and immunocompromised individuals [3]. Symptoms of NOE can vary widely but the most reported symptoms are otalgia, purulent otorrhea and hearing loss [4]. Patients can also present as systemically unwell with fever and chills [5,6]. The most commonly isolated pathogen in NOE is *Pseudomonas aeruginosa* but *Staphylococcus epidermidis*, *Proteus mirabilis* and fungal infections have also been identified. Otoscopy examination reveals oedema and granulation tissue, and the biopsy of external auditory canal can exclude carcinoma or other aetiologies [4]. Imaging studies are important for determining the presence of osteomyelitis, the extent of disease, and response to therapy. For an initial assessment of cranial osteomyelitis, CT and MRI imaging are both useful [3]. CT scanning remains the most important tool for diagnosis of skull base osteomyelitis (SBO) by demonstrating bone erosions, whereas MRI is more sensitive in detection of intracranial complications. ENT UK guidelines for clinically suspected NOE recommend urgent (two-week wait) referral for CT temporal bone, a baseline MRI head with contrast. Once the diagnosis is confirmed, it is treated with six weeks of antibiotics followed by a scheduled MRI after six weeks of discontinuation of antibiotics and regular monitoring of the disease for six months after treatment. 

Overall prognosis of necrotising otitis externa depends on multiple factors - extension of the disease and involvement of cranial nerves. It is estimated in a study that disease-related mortality was 7.7% [7] and in another 22% had failure of the treatment and relapse rate was 7% [8]. In a general overview, elderly patients with multiple comorbidities and reduced physiological reserve hold a guarded prognosis when encountered with NOE. 

## Case presentation

**Figure 1:**
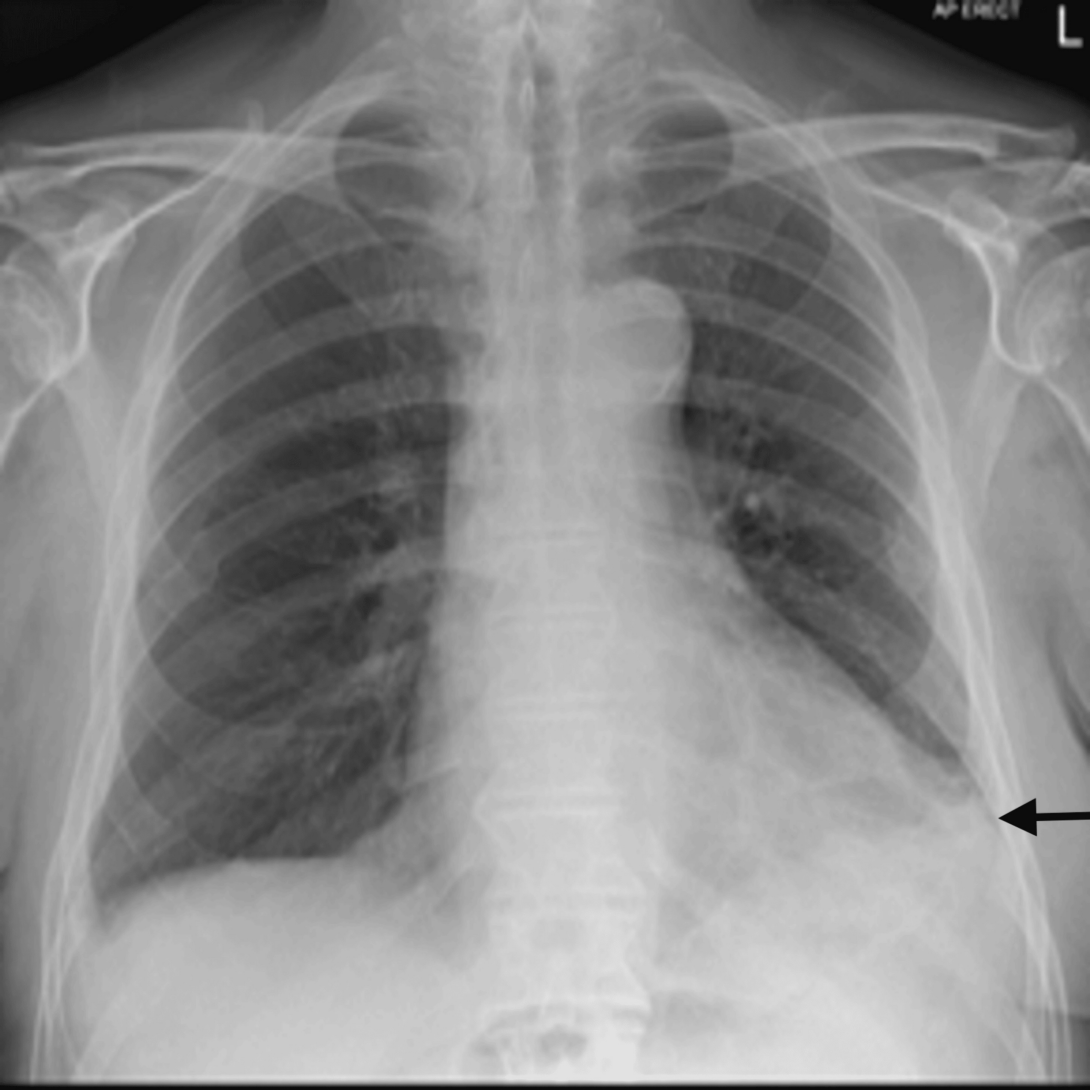
Chest X-ray: small left pleural effusion with left basal consolidation. This is an initial Chest X-ray at the time of admission to the hospital.

**Figure 2:**
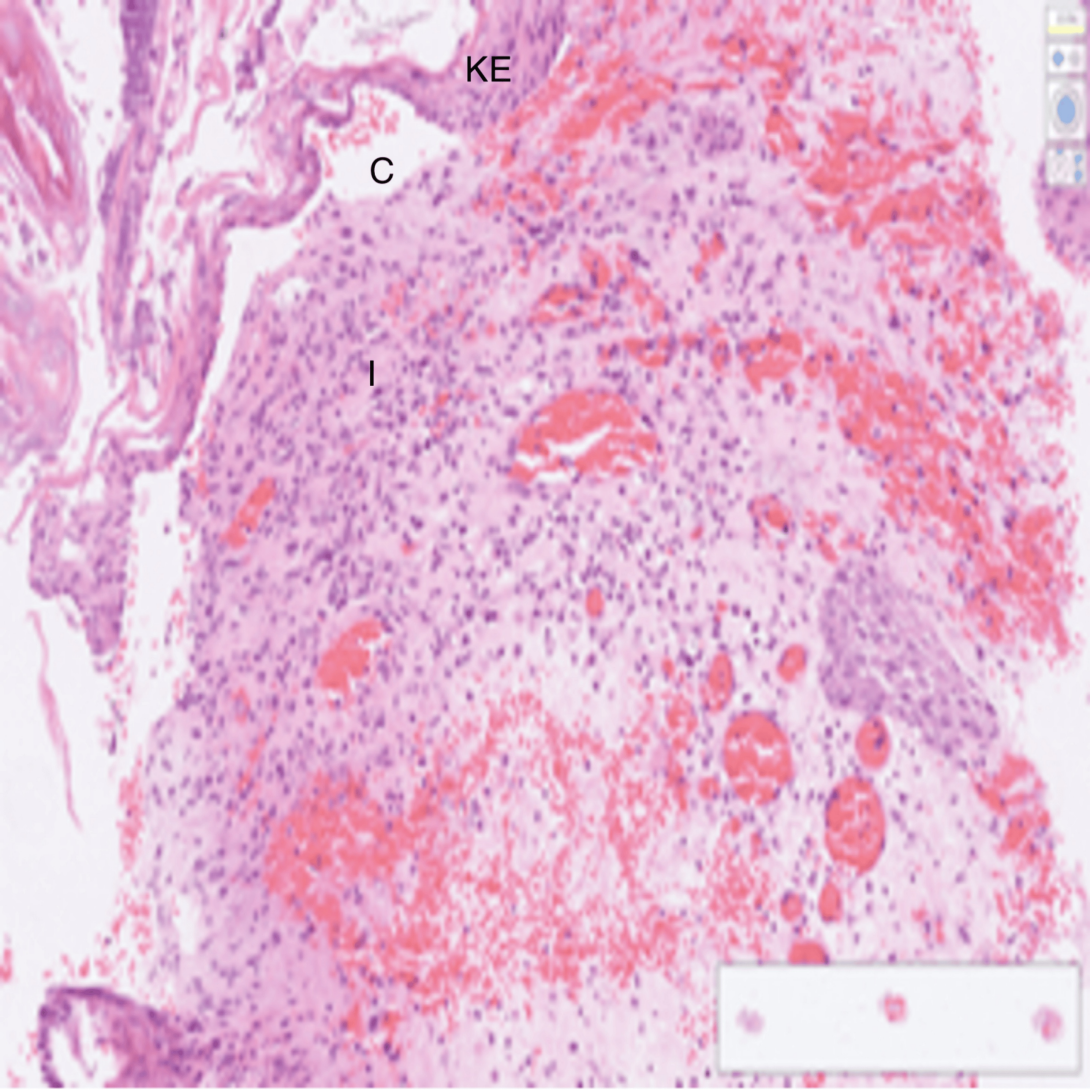
Cross-sectional preparation of the external ear shows keratinized stratified squamous epithelium (KE), underlying cartilaginous structure (C) and inflammatory cells (I). KE forms the outermost layer of the external ear canal and auricle. C - underneath the epithelium is the elastic cartilage, which gives the external ear its flexibility and resilience and also maintains shape. I - indicates inflammatory cells, which shows that an immune response has been generated, characterised by inflammatory cells

An 83-year-old white male, with a background of type II diabetes mellitus and bilateral hearing impairment, presented to the emergency department with productive cough, shortness of breath, pyrexia and generally unwell. He was admitted in the ward under the Care of the Elderly. The patient was reviewed in the ENT Clinic three months ago for worsening left-sided hearing loss, bilateral otalgia and otorrhea and was managed on the lines of bilateral otitis externa with topical and oral ciprofloxacin. An ear swab culture and a biopsy from the left ear polyp were taken for histology and microbiology. The left ear swab grew *Pseudomonas aeruginosa* and *Raoultella ornithinolytica*; polyp microscopy and histology showed mixed inflammatory cells compatible with an inflammatory aural/otic polyp with no signs of malignancy. After topical ciprofloxacin for seven days and oral ciprofloxacin 750 mg twice daily for two weeks, there was documented complete resolution of otalgia and inflammation on the right ear with residual signs of inflammation on the left ear. In one of the ENT visits, a diagnosis of grade 4 left Bell's palsy was made and he received a short course of oral steroid treatment with only mild improvement. An MRI Brain was planned and requested to exclude retrocochler lesion and NOE. However, the patient was admitted to hospital in the interim. During the hospital admission, blood tests (Table 1) showed WBC 13.8 X10^9^/l, neutrophils 12.7 X10^9^/l, and CRP 259 mg/L and chest X-ray confirmed left basal consolidation with small pleural effusion (Figure 1). Our initial diagnosis was community-acquired pneumonia, but in the view of dysphagia for liquids we also considered aspiration pneumonia as a differential diagnosis. An inpatient ENT review, along with ear swabs results of *Pseudomonas aeruginosa* and polyp biopsy showing inflammatory changes with no signs of malignancy (Figure 2), raised the suspicion of NOE.

**Table 1:** Laboratory results during the course of admission eGFR: estimated glomerular filtration rate, CRP: C-reactive protein, Hb: hemoglobin, Plt: platelets

Parameter	Day 1	Day 6	Day 12	Day 16	Day 18	Day 20	Reference Range
Sodium	135	141	143	147	154	144	133-146 mmol/L
Potassium	NA	NA	2.8	3.8	3.6	3.8	3.5-5.3 mmol/L
Urea	19.2	6.6	5.3	11.5	11.2	6.6	2.5-7.8 mmol/L
Creatinine	180	87	79	114	104	80	50-120 micromol/L
eGFR	29	71	79	51	56	77	>90 ml/min
CRP	259	120	74	132	127	69	<5 mg/L
WCC	13.8	7.9	7.7	9.2	6	3.5	4-11 x 10^9 /l
Hb	119	103	96	114	115	86	130-180 g/L
Plt	290	177	130	194	163	81	150-400 x 10^9/l

**Figure 3:**
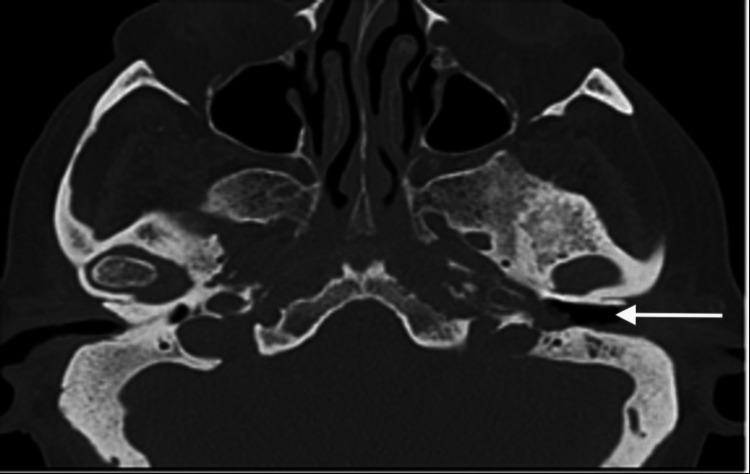
Plain CT head showing left skull base osteomyelitis, a characteristic finding in necrotizing otitis externa

**Figure 4:**
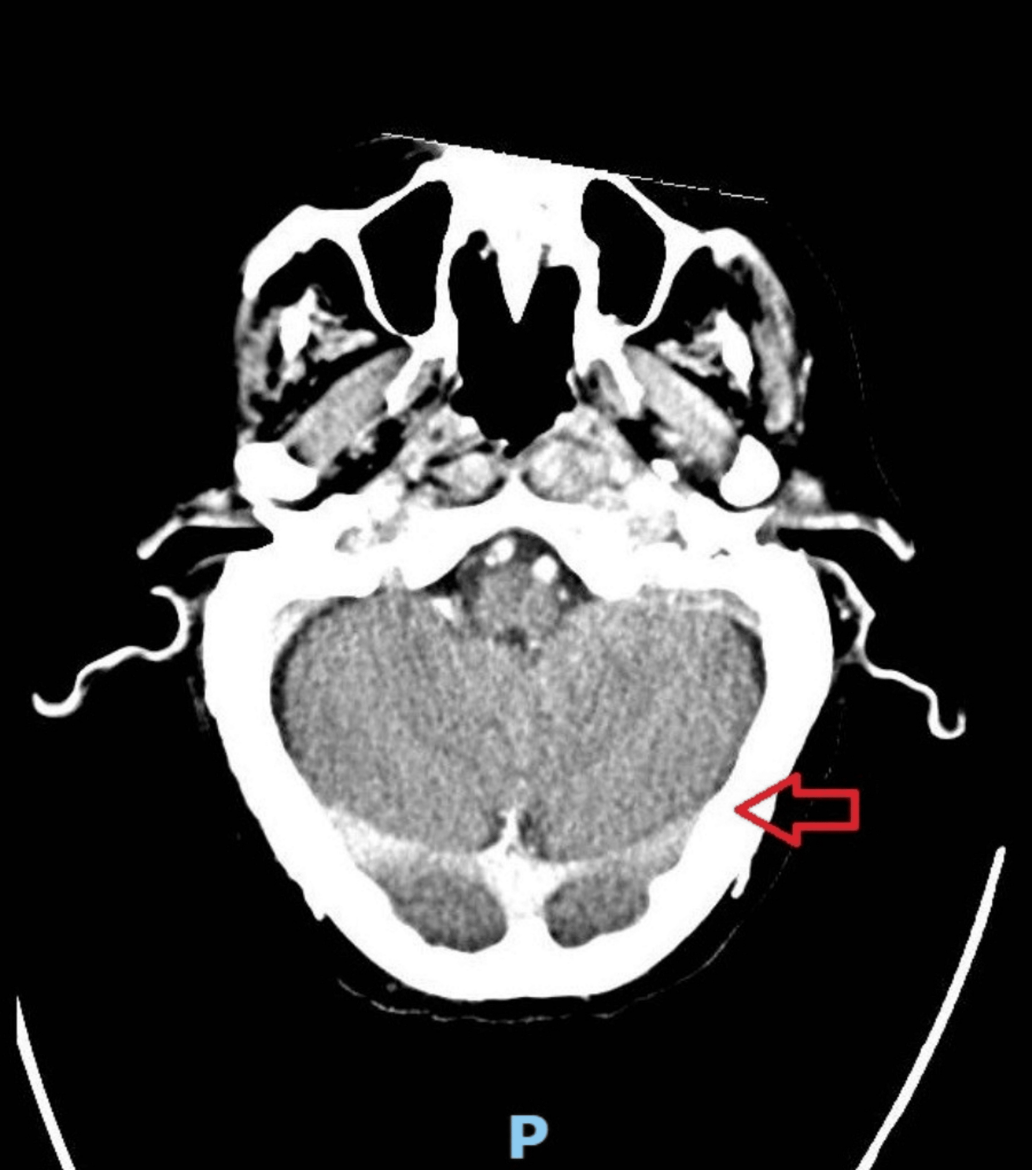
CT head; left sigmoid sinus thrombosis, secondary to the extension and progression of the disease

Neurological examination revealed unilateral signs of cranial neuropathy. A left-sided angle of mouth weakness, abnormal tongue movement and slurred speech were noticed, which are suggestive of cranial nerve XII palsy. A reduced palate movement on the left, decreased voluntary cough strength and mild hoarseness were also noticed, suggestive of nerve IX and X palsy. The patient had large variation in heart rate, respiratory rate and blood pressure and developed new onset of atrial flutter, probably caused by nerve X palsy. An urgent CT head revealed left-sided osteomyelitis of the temporal bone (Figure 3). He was started on an extended course of intravenous piperacillin/tazobactam and local aural ciprofloxacin drops for 10 days, but he had a poor clinical response to medical treatment. He further developed seizures and his level of consciousness decreased, which was indicative of signs of intracranial extension of the disease. A CT head with contrast was performed, which revealed left sigmoid sinus thrombosis and brain abscess (Figure 4). The patient was not fit for any surgical intervention due to limited physiological reserve and comorbidities. Despite intensive local and systemic antibiotics, the patient passed away in hospital.

## Discussion

There is a great variability in criteria for the diagnosis of NOE, which can result in delay in diagnosis [2]. Diagnosis of NOE is usually based on the presence of severe otalgia, otitis externa refractory to conventional treatment, diabetes mellitus or immunosuppressed status and confirmation of Pseudomonas on ear swab culture. The main patient complaint in this report was worsening left ear hearing compared with baseline rather than otalgia. The left hearing loss initially had a conductive component but a sensorineural component was added later on, suggesting nerve VIII (vestibulocochlear) palsy. The appearance of left facial palsy in this clinical context should be seen as refractory to treatment and progression of disease rather than an isolated idiopathic Bell's palsy. Presentation of NOE with dysphagia is also a rare occurence and, in this case, it was caused by SBO and involvement of lower cranial nerves, namely vagus (X) and hypoglossal (XII) [9].

This case is a presentation of NOE with multiple unilateral cranial neuropathies, namely, VII (facial), VIII (vestibulocochlear), IX (glossopharyngeal), X (vagus) and XII (hypoglossal). Traditionally, in NOE, the facial nerve (VII) is the most commonly affected but as the disease progresses, cranial nerves IX, X and XI (accessory) can be affected at the jugular foramen followed by hypoglossal nerve (XII) at the hypoglossal canal. Cranial nerves V (trigeminal) and VI (abducens) can also be affected if the disease extends to the petrous apex [10].

Despite unilateral neurological signs, which appeared in temporal relation with ear infection, there was lack of awareness of NOE until late in the course of the disease. Diagnosis was delayed as no otalgia was reported and he had almost normal otoscopy after completion of otitis externa treatment, except for inflammation in the left ear. Successive ENT reviews sustained the diagnosis of resolved bilateral otitis externa.

In this case, the CT head confirmed the diagnosis of NOE, with a delay of three months from the initial presentation due to atypical symptoms, such as hearing loss, dysphagia. In the current ENT UK Guidelines for NOE, there is recommendation for use of both CT and MRI in the diagnosis as well as in disease monitoring. It is recommended that cranial nerve palsies related to the jugular foramen should raise concern for sigmoid sinus thrombosis and intracranial complications such as meningitis or brain abscess. This patient developed sigmoid sinus thrombosis and brain abscess, which presented with decreased conscious level, seizures and was confirmed on the CT brain.

## Conclusions

Necrotizing otitis externa often presents as a diagnostic challenge. Facial palsy is a well-established late presentation of NOE but multiple cranial nerves involvement is a very rare presentation. Oropharyngeal dysphagia in the elderly should raise the suspicion of NOE, when other risk factors for this disease are present. Early diagnosis of NOE is based on high clinical suspicion but confirmation of disease is made radiologic by CT or MRI scans, which have synergistic roles. Clinical resolution of otitis externa can give a false reassuring if no radiological imaging is used.

The case highlights the importance of imaging in the diagnosis of NOE. In view of the overall adverse effects of NOE its early detection and treatment is paramount in the management and to prevent spread and complications such as seen in the aforementioned case - cranial nerve involvement and sigmoid sinus thrombosis.
